# Regulation of paxillin-p130-PI3K-AKT signaling axis by Src and PTPRT impacts colon tumorigenesis

**DOI:** 10.18632/oncotarget.10654

**Published:** 2016-07-18

**Authors:** Yiqing Zhao, Anthony Scott, Peng Zhang, Yujun Hao, Xiujing Feng, Saigopal Somasundaram, Ahmad M. Khalil, Joseph Willis, Zhenghe Wang

**Affiliations:** ^1^ Department of Genetics and Genome Sciences, Case Medical Center and Case Western Reserve University, Cleveland, Ohio 44106, USA; ^2^ Case Comprehensive Cancer Center, Case Western Reserve University, Cleveland, Ohio 44106, USA; ^3^ Department of Pathology, Case Medical Center and Case Western Reserve University, Cleveland, Ohio 44106, USA

**Keywords:** PTPRT, paxillin, Src, colorectal cancer

## Abstract

Protein tyrosine phosphatase receptor T (PTPRT) is frequently mutated in a variety of human cancers including colorectal cancer. Here we report that PTPRT knockout increases the size of mouse colon tumors in the Apc^min+/−^ genetic background, suggesting that inactivation of PTPRT promotes tumor progression. We previously demonstrated that PTPRT dephosphorylates paxillin at tyrosine-Y88 residue. Consistently, phosphorylation of Y88 paxillin (pY88) is up-regulated in colon tumors derived from Apc^min+/−^ Ptprt^−/−^ mice. An important downstream effector of pY88 paxillin is the oncogene Akt. Here, we show that pY88 paxillin impacts the Akt pathway by regulating the interaction between p130cas and the p85 regulatory subunit of PI3-Kinase. Additionally, while pY88 paxillin is a substrate of the tumor suppressor phosphatase PTPRT, the corresponding kinase has not been previously identified. In this study, we demonstrate that the oncogenic kinase Src directly phosphorylates paxillin at Y88. Moreover, colorectal cancer cells that express high levels of pY88 paxillin are sensitive to dasatinib treatment, suggesting that pY88 paxillin may serve as a predictive biomarker for Src family kinase inhibitors.

## INTRODUCTION

Protein tyrosine phosphorylation plays a critical role in virtually all human cellular processes that involve tumorigenesis [1]. Addition of phosphate to tyrosine of proteins by protein tyrosine kinases (PTKs) and its removal by protein tyrosine phosphatases (PTPs) are part of a collaborative process that controls functionally significant phospho-modification of important proteins that determine cancer cell phenotypes [2]. While the roles of PTKs in tumorigenesis are well documented, increasing evidence suggests that PTPs also play critical roles in cancer development [3]. In the first comprehensive mutational analysis of the entire PTP family in human cancers, we identified protein tyrosine phosphatase receptor-T (PTPRT) as the most frequently mutated PTP in colorectal cancers (CRC) [4]. A follow-up mutational analysis of PTP family genes in head and neck squamous cell carcinomas (HNSCC) also found that PTPRT is frequently mutated in this tumor type [5]. Moreover, recent whole-exome sequencing of various human cancers revealed that PTPRT is frequently mutated in a variety of human cancer types [6, 7, 8, 9, 10, 11]. A portion of PTPRT mutations are nonsense, insertion and deletion mutations resulting in premature truncation of the protein, whereas most of PTPRT mutations are missense. Our studies showed that the missense mutations located in the phosphatase domains reduce the phosphatase activity of PTPRT and that extracellular domain mutations impair its cell-cell adhesion ability [12–14]. Together, these data suggest PTPRT may normally function as a tumor suppressor. This notion is further supported by the study showing that PTPRT knockout mice are susceptible to azoxymethane (AOM)-induced colon tumor formation [15]. Apc^+/min^ mice provides a carcinogen-independent model of intestinal tumor. Here, we demonstrated that PTPRT knockout increases the size of colon tumors in Apc^+/min^ genetic background, suggesting that loss of PTPRT promotes tumor progression.

Given the compelling evidence showing that PTPRT functions as a tumor suppressor, it is important to understand the signaling pathways regulated by this phosphatase. To this end, using a phospho-proteomics approach, we identified and validated paxillin and STAT3 as the substrates of PTPRT [15, 16]. While PTPRT dephosphorylates the well-studied Y705 residue of STAT3 [16], the PTPRT target site on paxillin is a previously uncharacterized tyrosine-88 residue (paxillin Y88) [15]. We showed that PTPRT directly dephosphorylates pY88 paxillin [15]. Paxillin is an adapter protein involved in tumor growth, focal adhesion turnover, cell migration and metastasis [17–19]. It becomes tyrosine phosphorylated in response to external stimuli such as growth factors and adhesion to the extracellular matrix [20–22]. Paxillin Y88 is phosphorylated in response to PDGF-AA stimulation [15]. Using a knock-in method that targets endogenous gene loci [23], we changed the Y88 residue to phenylalanine (Y88F), a mutant that cannot be phosphorylated at this site. Paxillin Y88F mutant CRC cells displayed attenuated tumorigenicity, forming fewer colonies in soft agar and failing to form xenograft tumors in nude mice [15]. Moreover, compared to matched normal colon tissues, pY88 paxillin is up-regulated in a majority of human colon cancer specimens. In aggregate, these data suggest that pY88 paxillin plays an oncogenic role in colorectal tumorigenesis.

Given that PTPRT is a tumor suppressor, it would be challenging to target PTPRT mutations in cancers. However, the corresponding kinases of PTPRT substrates are potential therapeutic targets for patients whose cancers harbor PTPRT mutations. Here, we demonstrate that Src kinase directly phosphorylates Y88 paxillin. Furthermore, CRC cell lines expressing higher levels of pY88 paxillin are more sensitive to killing by Src inhibitor dasatinib, suggesting that pY88 paxillin may be exploited as a predictive biomarker for drugs targeting Src.

## RESULTS

### Ptprt KO increases the size of colon tumors in the Apc^min+/−^ genetic background

**Figure 1:**
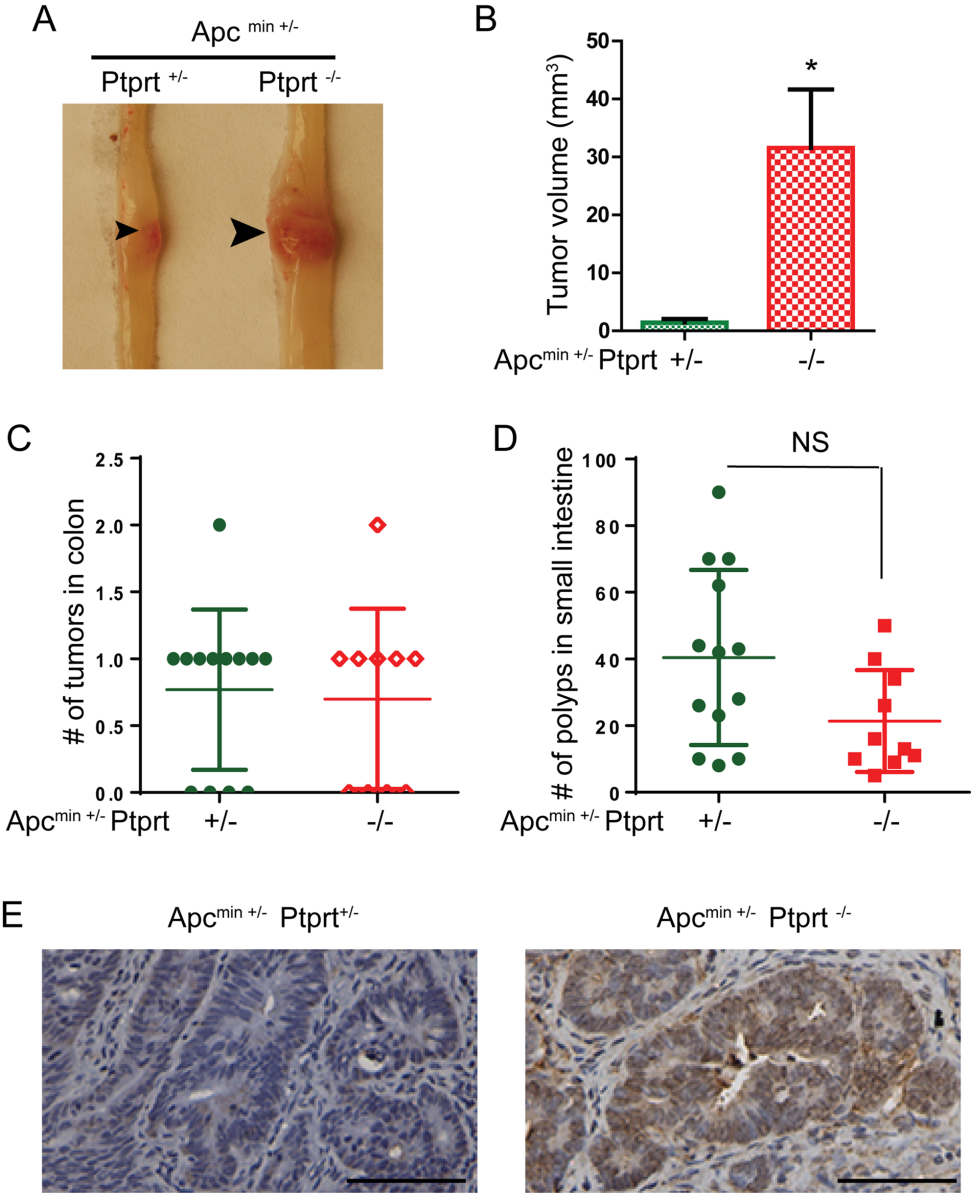
PTPRT knockout increases the size of colon tumors in Apc^+/min^ mice **A**. Representative image of colon tumors from Apc^+/min^ Ptprt^+/−^ and Apc^+/min^ Ptprt^−/−^ mice. Arrowheads indicate tumors. **B**. Average tumor size in the mouse colons of the indicated genotype. Apc^+/min^ Ptprt^+/−^ (n=10); Apc^+/min^ Ptprt^−/−^ (n=7). * p = 0.0026, t test. **C**. Number of colon tumors in mice of the indicated genotypes. **D**. Number of polyps in small intestine of mice with the indicated genotypes. NS, not significant, t test. **E**. Representative image of IHC staining of anti-pY88 paxillin antibody of colon tumors harvested from the indicated genotypes. Scale bar: 100 μm.

We previously demonstrated that Ptprt knockout (KO) mice were susceptible to AOM-induced colon tumor development [15]. To test if PTPRT knockout promotes colon tumorigenesis in a carcinogen-independent model, we bred Ptprt KO mice with Apc^min+/−^ mice, which are widely used to model human colon cancer. Remarkably, the sizes of the colon tumors developed in Apc^min+/−^ Ptprt^−/−^ mice were significantly larger than those in Apc^min+/−^ Ptprt^+/−^ mice (Figure 1A and 1B). However, the number of tumors in the two groups was similar, both in the colon and small intestine (Figure 1C and 1D). Given that PTPRT dephosphorylates paxillin at Y88 residue, we performed immunohistochemistry staining of colon tumors harvested from the mice. Compared to tumors in Apc^min+/−^ Ptprt^+/−^ mice, phospho-Y88 (pY88) paxillin is up-regulated in tumors in Apc^min+/−^ Ptprt^−/−^ mice (Figure 1E).

### Paxillin pY88 is up-regulated in primary, but not metastatic, CRCs

**Figure 2:**
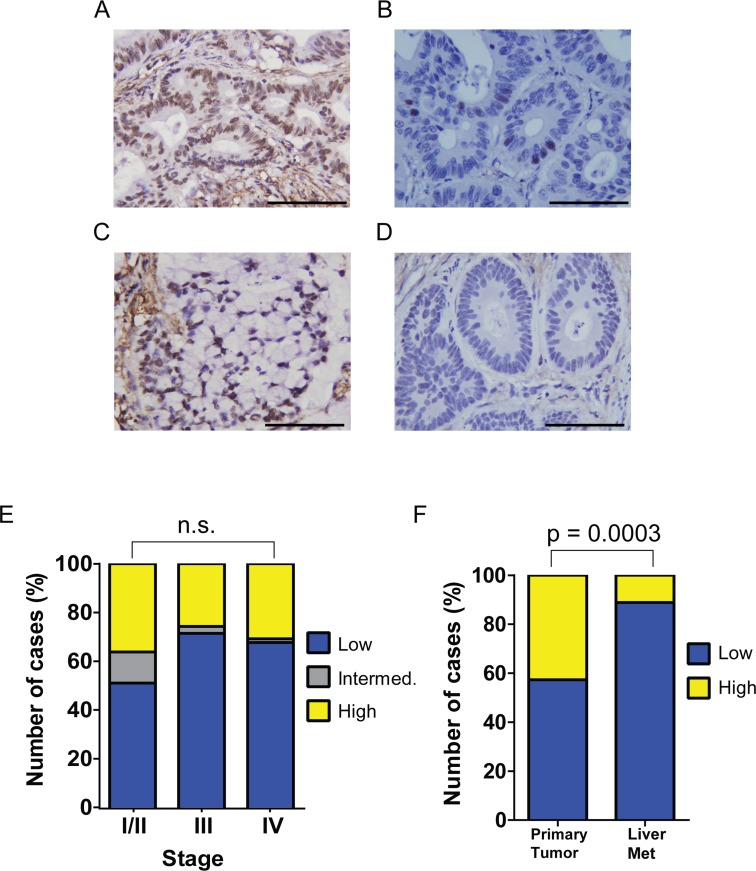
pY88 paxillin is upregulated in colorectal cancer tissues **A** to **D**. A representative image of strong pY88 paxillin immunohistochemical (IHC) staining of human colorectal carcinoma. pY88 paxillin high (A); low (B); signet ring CRC (C); no staining (D). Scale bar: 100 μm. **E**. Proportion of high, intermediate and low pY88 paxillin IHC staining in a tissue microarray sorted by tumor stage (n.s. – chi-squared test between Stage I/II and Stage IV, low vs. high staining). Stage I/II: n = 47; stage III: n = 35; stage IV: n= 148. **F**. Proportion of high and low pY88 paxillin IHC staining in a tissue microarray sorted by stage IV tumor site (primary versus liver) (p = 0.0003 – chi-squared test between primary tumor and liver metastasis, low vs. high staining). Primary stage IV tumors: n = 81; liver metastasis (liver met): n=67.

We have shown previously that pY88 paxillin is up-regulated in a majority of human colon cancer specimens compared to matched normal colon tissues [15]. To determine if varying levels of pY88 paxillin are associated with tumor prognosis, we stained a colorectal carcinoma tissue microarray with pY88 paxillin antibody for immunohistochemistry. Recorded patient characteristics include stage, age, right-sidedness of primary tumor, peritoneal involvement, good outcome, race, sex and microsatellite status (Table 1). Consistent with our initial report (13), we again observed strong pY88 paxillin staining in tumor tissue (Figure 2A to 2D). However, we were unable to correlate the staining intensity with stages (Figure 2E). We also tested tumors with signet ring morphology, as this subtype of colorectal carcinoma - although rare - is associated with worse outcomes [24]; pY88 paxillin staining did not correlate with this subtype. Intriguingly, there appears to be a relationship between Stage IV tumor location and pY88 paxilin staining. Tumors with strong pY88 paxillin staining tend to be primary Stage IV tumors (Figure 2F). Conversely, there are a greater proportion of liver metastases in the low paxillin pY88 cohort (Figure 2F), suggesting that tumor microenvironment may modulate paxillin Y88 phosphorylation.

**Table 1:** Patient characteristics

Clinical FeaturesAverage Age	High (n = 66)67.1	Low (n = 137)64.7
	%	%
**Right-sided**	63.2	72.5
**Peritoneal Involvement**	6.1	12.4
**Liver tumors**	7.5	29.0
**Good Outcome**	30.3	18.2
**Race - Black**	20.3	28
**Female Gender**	52	53
**Microsatellite unstable**	3.8	5.3
**Tumor stage**		
** I/II**	25.8	17.5
** III**	13.6	18.2
** IV**	60.6	64.2

### Src regulates paxillin Y88 phosphorylation

**Figure 3:**
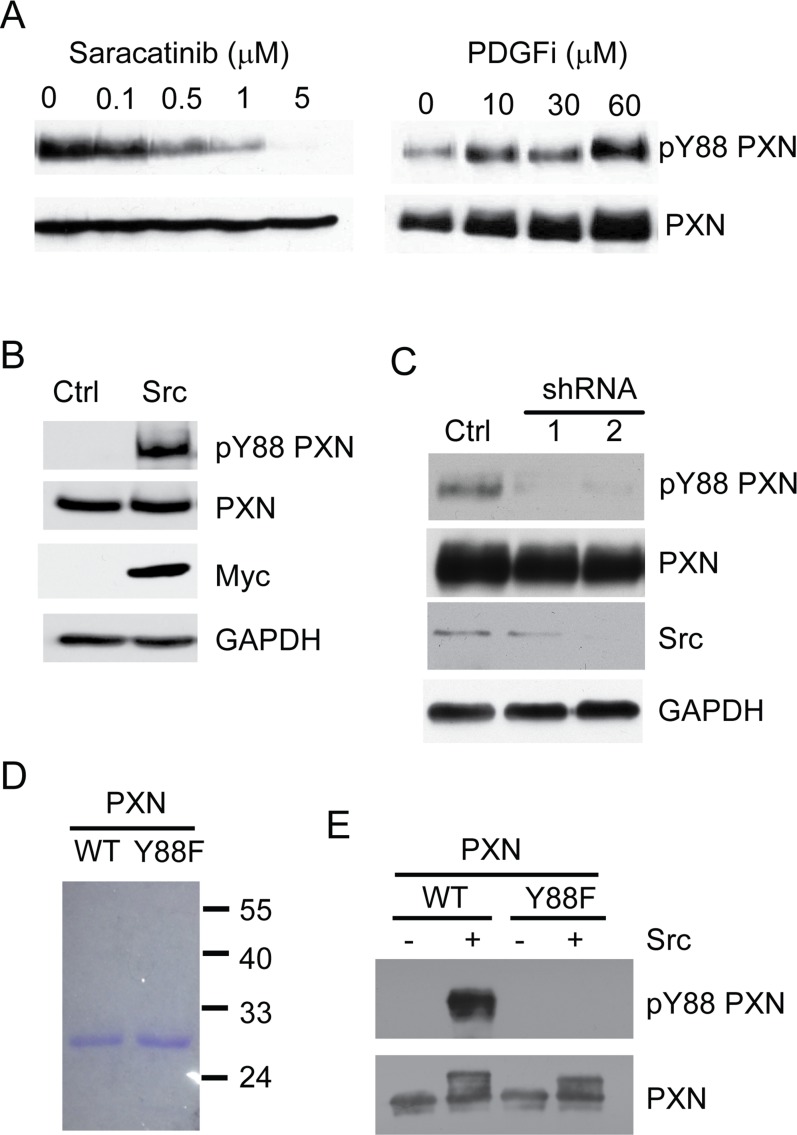
Src directly phosphorylates paxillin at Y88 residue **A**. Small molecule inhibitors of Src decrease pY88 paxillin (PXN) levels. HT29 CRC cells were treated with saracatinib (Src-specific kinase inhibitor) or imatinib mesylate (PDGFR-inhibitor control). Cells were lysed and the lysates blotted with the indicated antibodies. **B**. Overexpression of Src increases pY88 paxillin levels. HEK293 cells were transfected with a vector overexpressing Src or an empty control vector. Cells were lysed and the lysates blotted with the indicated antibodies. **C**. Knockdown of Src decreases pY88 paxillin levels. CRC cells were transfected with a shRNA vector against Src or a scrambled control vector. Cells were lysed and the lysates blotted with the indicated antibodies. **D**. Recombinant paxillin was expressed in bacteria, purified and resolved using SDS-PAGE. **E**. Equal amounts of paxillin and paxillin Y88F protein was mixed with recombinant Src, ATP and kinase assay buffer for the *in vitro* kinase assay. The reaction mixture was blotted with the indicated antibodies.

PTPRT dephosphorylates pY88 paxillin, but the kinase directly responsible for the reciprocal phosphorylation event at this site has not been previously identified. We identified the oncogenic kinase Src and PDGF receptor as potential kinases that phosphorylate the paxillin Y88 residue using a kinase prediction software [25]. Therefore, we set out to test whether inhibitors against these two tyrosine kinases could reduce pY88 paxillin levels in CRC cells. As shown in Figure 3A, saracatinib, a Src-specific kinase inhibitor, decreased pY88 paxillin phosphorylation in a dose dependent manner in HT29 CRC cells. A PDGFR inhibitor imatinib (Gleevec^®^) had no effect on paxillin Y88 phosphorylation (Figure 3A). Consistently, Src overexpression caused pY88 paxillin levels to increase dramatically (Figure 3B). Conversely, knocking down Src using shRNA attenuated pY88 paxillin levels (Figure 3C). Taken together, our data suggest that Src is a kinase that phosphorylates paxillin Y88.

### Src directly phosphorylates paxillin at Y88

We next tested if Src phosphorylates paxillin Y88 *in vitro*. A 6xHistidine tagged N-terminus paxillin fragment (amino acids 1 to 165) was expressed in E. coli and purified to near homogeneity (Figure 3D). The purified recombinant paxillin proteins were incubated with purified and active recombinant Src proteins in the presence of ATP. Phospho-Y88 paxillin was detected by Western blot analyses using a phospho-specific antibody recognizing pY88 paxillin. Figure 3E shows that Src indeed phosphorylated paxillin at the Y88 residue *in vitro*. In contrast, the anti-pY88 paxillin antibody failed to detect any signal in a control experiment using a recombinant paxillin Y88F mutant protein as a substrate, demonstrating the specificity of the antibody. Notably, incubation of Src with paxillin Y88F mutant protein induced a mobility shift of the paxillin fragment, suggesting that Src is capable of phosphorylating other tyrosine residues in the N-terminal fragment of paxillin. Nonetheless, our data demonstrate that Src directly phosphorylates paxillin Y88.

### Paxillin pY88 may serve as a predictive factor for dasatinib sensitivity

**Figure 4:**
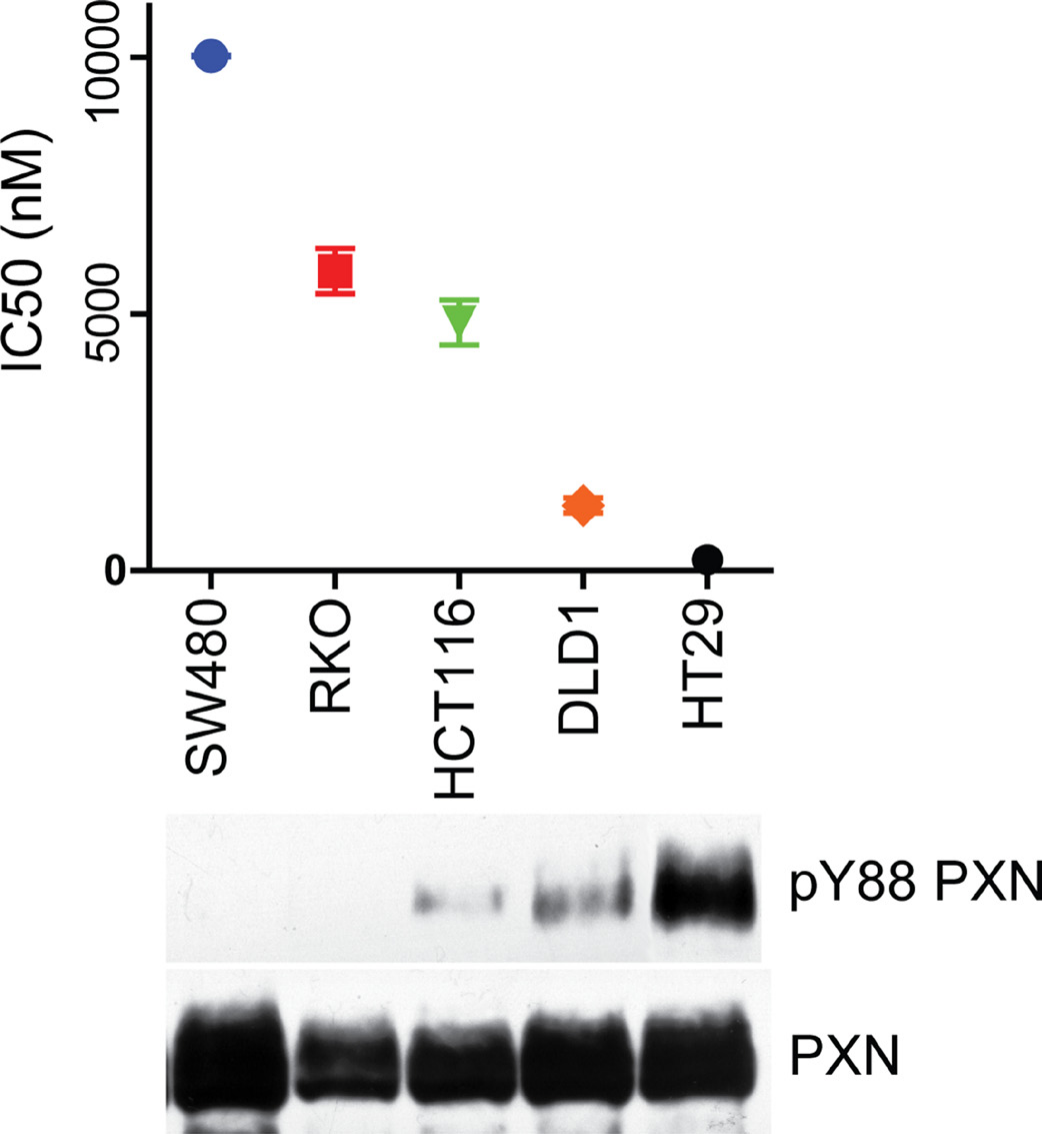
pY88 paxillin levels predict sensitivity to dasatinib A panel of CRC cell lines was treated with varying concentrations of dasatinib and viable cells were counted using a cell counting kit. Percent of viable cells were plotted with drug concentration using the Prism Graphpad software and the IC_50_ of these lines was calculated. The indicated cells were grown without dasatinib and the lysates blotted with the indicated antibodies.

Although pY88 paxillin immunohistochemistry does not have prognostic value, we wanted to see if it could predict dasatinib sensitivity. Dasatinib, a Src family kinase inhibitor used to treat acute lymphoblastic and chronic myelogenous leukemias [26], is currently in clinical trials in conjunction with standard treatment protocols for advanced metastatic colorectal cancer [27, 28]. Given that we demonstrated that Src is the kinase that phosphorylates paxillin Y88, we tested the Src family kinase inhibitor dasatinib on a panel of CRC cell lines with varying levels of pY88 paxillin. Cell lines with low levels of pY88 paxillin were resistant to dasatinib treatment, indicated by a high IC_50_ value (Figure 4). Conversely, cell lines with progressively higher levels of pY88 paxillin levels had low IC_50_ values and thus are more sensitive to dasatinib (Figure 4). Therefore, our data suggest that pY88 paxillin may be exploited as a predictive marker for response of colorectal cancer patients to dasatinib.

### pY88 paxillin regulates PI3-Kinase activation

**Figure 5:**
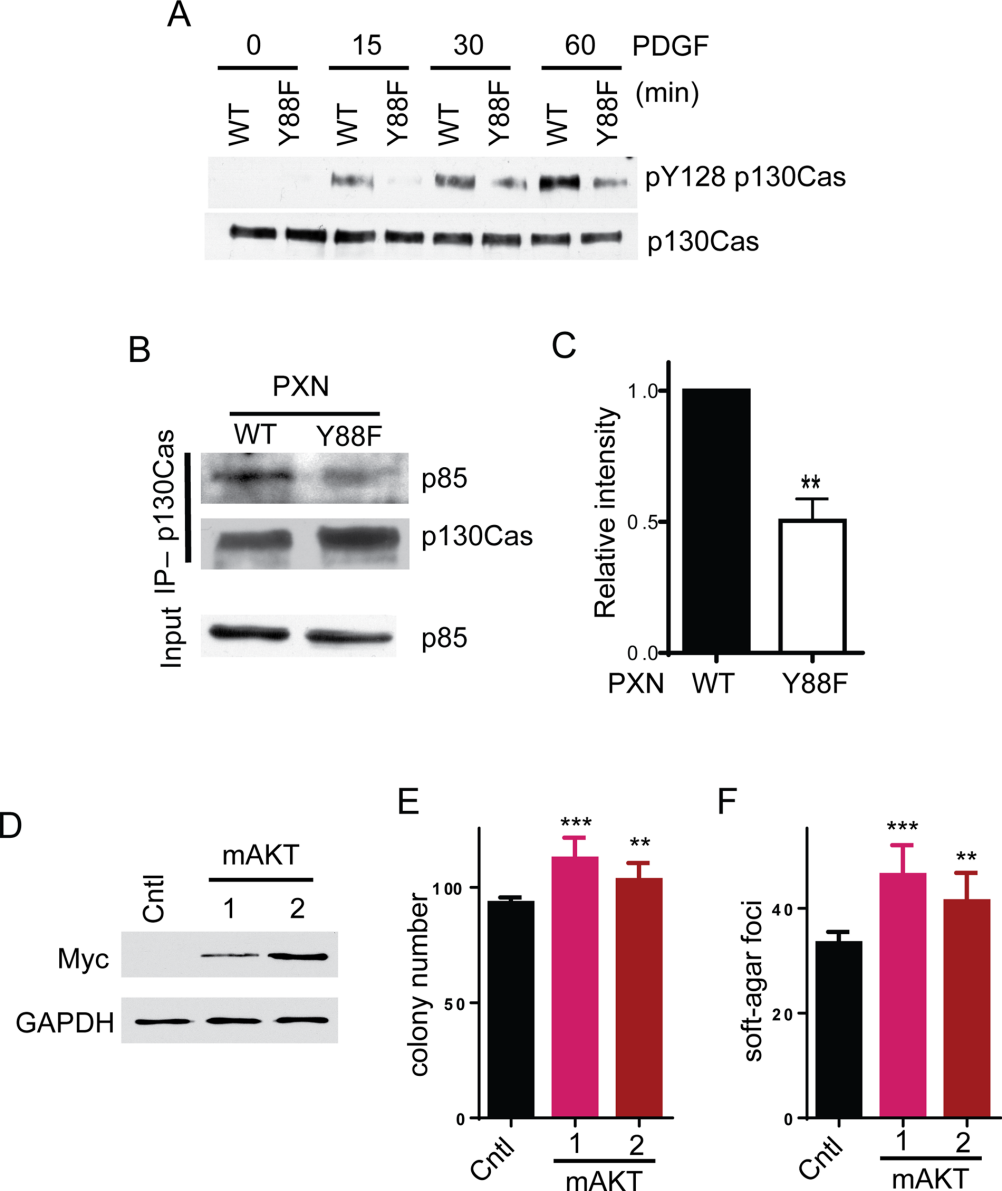
pY88 paxillin activates p130cas-PI3K-AKT signaling axis **A**. Parental (WT) and paxillin Y88F cell lines were starved overnight and stimulated with PDGF-AA for the indicated times. Cell lysates were blotted with the indicated antibodies. **B**. HCT116 parental cells or HCT116 paxillin Y88F cell lines were lysed and the lysates were immunoprecipitated with anti-p130cas antibody. The immunocomplexes and lysates were blotted with the indicated antibodies. **C**. Quantified p85 signal intensity was averaged over four independent replicates of this experiment using ImageJ software (** *p* = 0.001, *t* test). **D** to **F**. Expression of myristoylated AKT (mAKT) increases colony and soft-agar foci formation of paxillin Y88F mutant cells. HCT116 paxillin Y88F homozygous mutant cells were transfected with either empty vector as a control or myc-tagged mAKT1. A control clone and two stable clones expressing mAKT were selected. Cell lysates of the indicated clones were blotted with the indicated antibodies (D). The indicated clones were grown on plastic for colony numbers were counted (E). The indicated clones were grown in soft agar and soft agar foci were counted (F).

**Figure 6:**
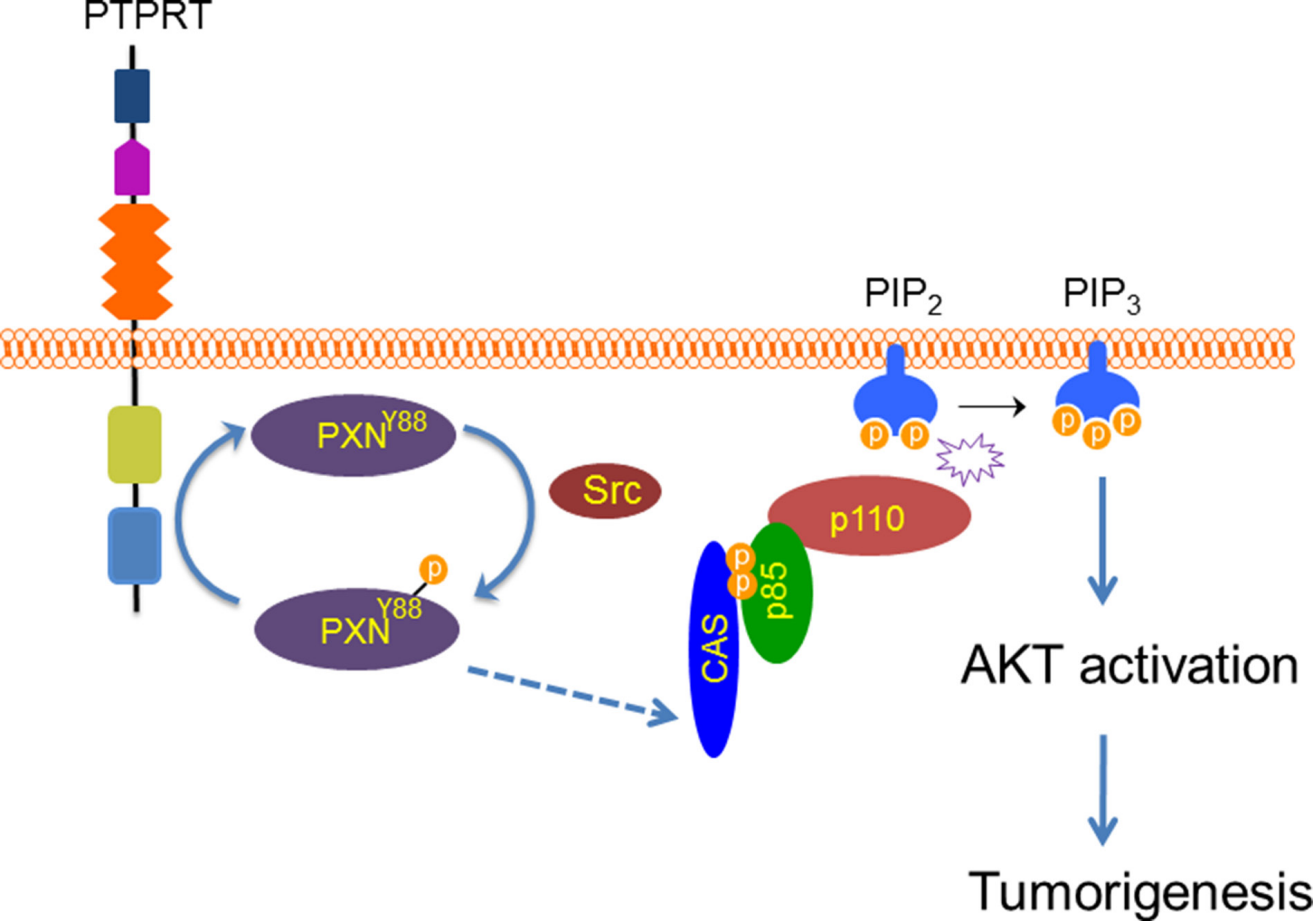
A model of phosphorylation of Y88 paxillin by Src leading to activation of AKT Paxillin becomes phosphorylated by Src at Y88. In turn, p130cas phosphorylation is upregulated, potentiating its interaction with p85 (dotted line indicates undetermined steps). p130cas-p85 interaction activates the p110 subunit of PI3-kinase and AKT, which then leads to cellular transformation.

Previous studies show that phospho-Akt is a major downstream effector of pY88 paxillin, as Akt phosphorylation induced by PDGF is significantly attenuated in paxillin Y88F mutants [15]. How this signal is transduced has not yet been determined. It is well documented that AKTs are activated by phosphatidylinositol 3-kinase (PI3K) [29]. PI3K converts phosphatidylinositol 4,5-bisphosphate (PIP2) to phosphatidylinositol 3,4,5-trisphosphate (PIP3). PIP3 then recruits AKT and PDK1 to the plasma membrane. There PDK1 phosphorylates and activates AKT. PI3K consists of a p85 regulatory subunit and a p110 catalytic subunit. PI3K becomes activated when it is recruited to the membrane by interaction between p85 and phospho-tyrosine residues on membrane-bound receptors or adapter proteins [29]. Several studies demonstrated that membrane-associated p130cas activates PI3K through interaction with p85 and that this interaction is dependent on tyrosine phosphorylation of the substrate-binding domain on p130cas [30–32]. We previously showed that paxillin pY88 regulates p130cas tyrosine phosphorylation at tyrosine-165 (Y165), located in the substrate domain that will engage p85 [15]. Recent evidence has also implicated phosphorylation of p130cas tyrosine-128 (pY128), another tyrosine residue located in the substrate-binding domain on p130cas, in PI3K-Akt regulation [32]. Consistent with pY165 p130cas, parental cells demonstrate robust phosphorylation of pY128 p130cas post-PDGF stimulation. However, this phosphorylation event is dramatically attenuated in paxillin Y88F cells (Figure 5A). We thus hypothesized that p130cas protein in paxillin Y88F mutant cells fails to interact with p85, or it does so to a lesser extent, thereby resulting in reduced PI3K and AKT activation. As shown in Figure 5B and 5C, p130cas binds p85 more tightly in parental cells versus paxillin Y88F cell lines, suggesting that pY paxillin transduces signal to enhance recruitment of PI3K to p130Cas, thereby activating that PI3K-AKT pathway. Moreover, expression of a constitutively active form of AKT (myristoylated AKT) increased colony and soft-agar foci formation of paxillin Y88F homozygous mutant cells (Figure 5D to 5F). Together, our data suggest that phosphorylation of paxillin Y88 activates AKT through the paxillin-p130Cas-p85/PI3K-AKT signaling axis and promotes colorectal tumorigenesis (Figure 6).

## DISCUSSION

Using Apc^+/min^ mouse model, we demonstrate here that knockout of PTPRT increases the size of colon tumors, suggesting that loss of PTPRT promotes tumor progression. Consistent with our previous observation [15], knockout of PTPRT leads to increased levels of pY88 paxillin in colon tumors. The tumor suppressor PTPRT is mutated in a variety of cancers and as such is an appealing potential therapeutic target. However, given that PTPRT normally functions as a tumor suppressor and that its function is lost in cancer, it would be extremely difficult to reactivate PTPRT function in tumors. Therefore, it is crucial to identify the agent responsible for the corresponding oncogenic event; in the case of PTPRT, the kinase that phosphorylates its phospho-tyrosine substrate. Here we show that Src kinase phosphorylates paxillin Y88, the target site of PTPRT. Most importantly, we showed that CRC cells expressing high levels of pY88 paxillin are sensitive to Src kinase inhibition, suggesting that this phosphorylation event may be exploited as a predictive biomarker for Src family kinase inhibitors. However, further *in vivo* study in a xenograft and/or human clinical trials is needed to validate this observation.

Although a previous study identified Y88 on paxillin as a possible site for phosphorylation, it was largely thought to be a minor event and of no physiologic importance [21]. In contrast, prior work from our laboratory [15] and this study strongly suggest otherwise. The decreased tumorigenicity of paxillin Y88F mutants described previously [15] indicates this phosphorylation event is important in cancer development. Moreover, down-regulation of pY88 paxillin by Src inhibitor data and shRNA suggests that paxillin Y88 is a physiologic substrate of Src. Using a specific pY88 paxillin antibody, we prove that Src directly phosphorylates pY88 paxillin by an *in vitro* kinase assay. Taken together, our study demonstrates unequivocally that Src is a kinase that directly phosphorylates paxillin at Y88.

Additionally, we investigated further the *in vivo* pattern of pY88 paxillin levels. We showed previously that it is upregulated in tumors versus normal matched controls [15], but it is not associated with disease prognosis. However, in metastatic liver tumors, pY88 paxillin levels are dramatically reduced. This finding suggests that phosphorylation of paxillin Y88 is strongly affected by the tumor microenvironment.

In this study, we also show how pY88 paxillin transduces a signal to activate Akt, an important mechanism for oncogenic growth. PI3K is activated by engagement with phospho-tyrosine substrates. One such SH2-domain containing protein is the p85 subunit of PI3K, as its docking with tyrosine-phosphorylated p130cas activates the p110alpha subunit [30–32]. This interaction is attenuated in paxillin Y88F mutants, suggesting pY88 paxillin potentiates PI3K-AKT signaling via p130cas-p85 interaction. However, an important outstanding question from this study is how pY88 paxillin upregulates the phosphorylation of p130cas. Specifically, tyrosine-165 and tyrosine-128 on p130cas both are phosphorylated to a greater extent in parental versus paxillin Y88F mutant cells, but other phosphorylation targets on p130cas (such as tyrosine-249 and tyrosine-410) are unaffected by the paxillin Y88F mutation [15]. Given this lead, further research in how pY88 paxillin affects kinases, phosphatases and signaling molecules will improve our knowledge how it impacts *in vivo* tumorigenesis.

## MATERIALS AND METHODS

### Mice

Animal experiments were approved by the Case Western Reserve University Animal Care and Use Committee. PTPRT KO mice were generated as described previously [15]. C57/bl6 Ptprt^+/−^ mice were crossed with Apc^+/min^ mice to generate mice of the experimental group. Mice were sacrificed at 3 month-old to examine tumor formation in small intestine and colon. Size of tumors were calculated as (length x width^2^)/2. Colon tumors were fixed with formalin and embedded for H&E and IHC staining.

### Cell culture and reagents

HCT116, DLD1, HT29, SW480, RKO and HEK 293 cells were obtained from the American Type Culture collection (Manassas, VA, USA). HEK 293 cells were grown in DMEM + 10% FBS while the other cell lines were grown in McCoy's 5A +10% FBS. Paxillin Y88F homozygous knock-in cell lines were generated in DLD1 and HCT116 parental cells as described previously [15]. For co-immunoprecipitation studies, cells were starved overnight and stimulated with 30 ng/ml of platelet-derived growth factor-AA (PDGF-AA) for one hour.

### Transfection

Cells were transfected using Lipofectamine transfection reagent (Invitrogen) as previously described [33]. After 48 hours, cells were either lysed (transient transfection) or put into selective media (stable transfection). The Src overexpression insert used a pCMV-3Tag-2A-3xmyc backbone (Aglient Technologies, Santa Clara, CA, USA). Src shRNA knockdown used the Mission pLKO.1-puro system (Sigma-Aldrich, St. Louis, MO, USA).

### *In vitro* kinase assay

The N-terminal 165 amino acids of paxillin were cloned into the pET28 vector and expressed in BL21 competent bacteria. Protein was purified using the Qiaexpressionist (Qiagen, Valencia, CA, USA) protocol. Briefly, expression was induced overnight using 0.1mM IPTG, cells were lysed and protein was purified from lysate using Ni-NTA agarose. Purified protein was then dialyzed using a slide-a-lyzer dialysis cassette (Thermo Fisher Scientific, Waltham, MA, USA). Dialyzed protein was added to a reaction mixture containing ATP, buffer and recombinant Src (Promega, Fitchburg, WI, USA) and the reaction proceeded for 1h at room temperature.

### Cell lysis

Cells were either lysed using a urea lysis buffer (10 mM Tris-HCl, pH 8.0; 100 mM NaH_2_PO_4_; 8 M urea; 1mM Na_3_VO_4_; 20mM NaF; 80 μM beta-glycerophosphate; 20 mM sodium pyrophosphate) or a Flag lysis buffer (50 mM Tris-HCl pH 7.5; 1 mM EDTA; 150mM NaCl; 1% Triton X-100; 1 mM Na_3_VO_4_; 20 mM NaF; Protease Inhibitor Cocktail (Roche, Penzberg, GER) for co-immunoprecipitation. Western blots were performed as described previously [15]. Antibodies include anti-pY88 paxillin, anti-PI3K p85 (Millipore, Temecula, CA, USA) anti-Src (Cell Signaling, Danvers, MA, USA), anti-paxillin, anti-p130cas (BD Biosciences, San Jose, CA, USA), anti-GAPDH (Santa Cruz Biotechnology, Santa Cruz, CA, USA).

### Immunohistochemistry

Paraffin-embedded human tissue was deparaffinized in xylene and boiled for 20 minutes for antigen retrieval. Samples were incubated in primary pY88 paxillin antibody overnight. The sections were stained with secondary antibody for 30 min at room temperature then visualized with EnVision-HRP kit (Dako). The immunohistochemical staining was reviewed blindly by a board-certified pathologist (Dr. Willis). Stained sections were classified according to the intensity of staining and the percentage of cells showing paxillin Y88 phosphorylation staining. This was assessed in a semi-quantitative manner with assignment of staining ranging from 0 to 4+. Tumors with a score of 2+ and above are defined as high pY88 paxillin staining.

### Colony formation and focus formation in soft agar

For colony formation, cell lines were placed into 6-well plates at 200 cells per well. Cells were grown for 14 days before staining with crystal violet (Sigma, St. Louis, MO). For the soft agar assay, cell lines were plated at 1000 cells/ml in top plugs consisting of 0.4% SeaPlaque agarose (FMC Bioproducts, Rockland, ME) and McCoy's 5A medium. After 30 days, the colonies were photographed and counted.

### Statistical analysis

We applied the *t* test to compare the means between two groups, assuming unequal variances. For immunohistochemistry, chi-square tests were used.
